# Kidney-type glutaminase is a biomarker for the diagnosis and prognosis of hepatocellular carcinoma: a prospective study

**DOI:** 10.1186/s12885-023-11601-y

**Published:** 2023-11-09

**Authors:** Laizhu Zhang, Ke Su, Qi Liu, Binghua Li, Ye Wang, Chunxiao Cheng, Yunzheng Li, Chun Xu, Jun Chen, Hongyan Wu, Mengxia Zhu, Xiaoli Mai, Yajuan Cao, Jin Peng, Yang Yue, Yitao Ding, Decai Yu

**Affiliations:** 1 Division of Hepatobiliary and Transplantation Surgery, Department of General Surgery, Nanjing Drum Tower Hospital, Affiliated Hospital of Medical School, Nanjing University, Nanjing, China; 2 Nanjing Drum Tower Hospital Clinical College of Nanjing University of Chinese Medicine, Nanjing, China; 3 Department of Pathology, Nanjing Drum Tower Hospital, Affiliated Hospital of Medical School, Nanjing University, Nanjing, China; 4 Department of Radiology, Nanjing Drum Tower Clinical Medical School, the Affiliated Hospital of Nanjing Medical University, Nanjing, China

**Keywords:** Biomarker, Diagnosis, Glutaminase, Hepatocellular carcinoma, Prognosis

## Abstract

**Purpose:**

The pathological diagnosis and prognosis prediction of hepatocellular carcinoma (HCC) is challenging due to the lack of specific biomarkers. This study aimed to validate the diagnostic and prognostic efficiency of Kidney-type glutaminase (GLS1) for HCC in prospective cohorts with a large sample size.

**Methods:**

A total of 1140 HCC patients were enrolled in our **prospective** clinical trials. Control cases included 114 nontumour tissues. The registered clinical trial (ChiCTR-DDT-14,005,102, *chictr.org.cn*) was referred to for the exact protocol. GLS1 immunohistochemistry was performed on the whole tumour section. The diagnostic and prognostic performances of GLS1 was evaluated by the receiver operating characteristic curve and Cox regression model.

**Results:**

The sensitivity, specificity, positive predictive value, negative predictive value, Youden index, and area under the curve of GLS1 for the diagnosis of HCC were 0.746, 0.842, 0.979, 0.249, 0.588, and 0.814, respectively, which could be increased to 0.846, 0.886, 0.987,0.366, 0.732, and 0.921 when combined with glypican 3 (GPC3) and alpha-fetoprotein (AFP), indicating better diagnostic performance. Further, we developed a nomogram with GPC3 and GLS1 for identifying HCC which showed good discrimination and calibration. GLS1 expression was also related with age, T stage, TNM stage, Edmondson–Steiner grade, microvascular invasion, Ki67, VEGFR2, GPC3, and AFP expression in HCC. GLS1 expression was negatively correlated with disease-free survival (*P* < 0.001) probability of patients with HCC.

**Conclusions:**

It was validated that GLS1 was a sensitive and specific biomarker for pathological diagnosis of HCC and had prognostic value, thus having practical value for clinical application.

**Supplementary Information:**

The online version contains supplementary material available at 10.1186/s12885-023-11601-y.

## Introduction

Liver cancer was the sixth most prevalent cancer and the fourth leading cause of cancer death worldwide in 2018 [1]. The prevalence of liver cancer in China was 9.6%, with a crude mortality rate of 13.9%. Additionally, the prognosis of liver cancer still was the poorest while the 5-year survival rate of cancers has greatly increased in recent years [2]. As the most common type of liver cancer, hepatocellular carcinoma (HCC) has several causes, such as hepatitis virus, nonalcoholic fatty liver disease, alcoholic cirrhosis, and so forth [3, 4]. In China, hepatitis B virus infection is the main reason for HCC. The precise identification of early-stage HCC from cirrhotic lesions and dysplastic nodules is a key step for the patients to receive timely and correct treatment, which can help achieve long-term survival.

Some studies have confirmed that the traditional biomarkers have high specificity but less-than-perfect sensitivity even if they are combined, such as glypican 3 (GPC3), glutamine synthetase, alpha-fetoprotein (AFP), heat shock protein 70 (HSP 70), and so forth [5–7]. Although they has some efficacy in diagnosing HCC, currently, not many effective biomarkers exist for predicting prognosis in patients with HCC.

Given that the metabolic reprogramming of tumours is a hallmark of cancer, one important process is glutaminolysis [8]. GLS1 can decompose glutamine, produce glutamate, and participate in biosynthesis, energy metabolism, and oxidative stress [9]. The expression of glutaminase proteins, which regulates the first stage in glutamine metabolism, is associated with the malignancy and pace of cancer progression [10, 11]. GLS1 promotes proliferation and compromises tumour growth when it is suppressed or knocked down [12, 13]. In our previous study, it was found that GLS1 was highly expressed in tumour tissues, and the expression gradually increased from low-grade dysplastic nodules, to high-grade dysplastic nodules, to early- and advanced-stage HCC [14]. Also, the grade of the tumour histology is correlated with its increased expression [12, 15]. It has high sensitivity and specificity in diagnosing HCC and is negatively associated with prognosis. Furthermore, the expression of GLS1 is correlated with serum AFP level, tumour differentiation, lymphatic metastasis, and TNM stage in patients with HCC [13]. Our further investigations have shown that targeting glutaminase 1 reduces the stemness characteristics of HCC by upregulating reactive oxygen species and downregulating the Wnt/beta-catenin pathway [12, 14]. Hence, GLS1 plays a significant role in the occurrence and development of HCC. It can provide certain reference information for diagnosis or prognosis in clinical practice.

Based on our previous research, we designed a prospective clinical trial to further validate the diagnostic efficacy of GLS1 for HCC with larger sample size. At the same time, it was hypothesized that GLS1 was associated with tumour biological behaviour and disease-free survival (DFS) in patients with HCC.

## Materials and methods

### Patients

From January 1, 2016 to December 31, 2020, the data of patients who underwent radical surgery or liver biopsy at our center were selected. The inclusion criteria were as follows: (1) primary HCC with no preoperative treatment, such as ablation, transarterial chemoembolization, chemotherapy, immunotherapy, or targeted therapy; (2) HCC confirmed by postoperative pathology according to the gold standard: histomorphology (hematoxylin and eosin staining) and traditional immunohistochemical biomarkers; (3) had immunohistochemical results for AFP, GLS1, and GPC3 simultaneously; and (4) surgical specimens having completely pathological data including all parameters explored in this study.

**Fig. 1:**
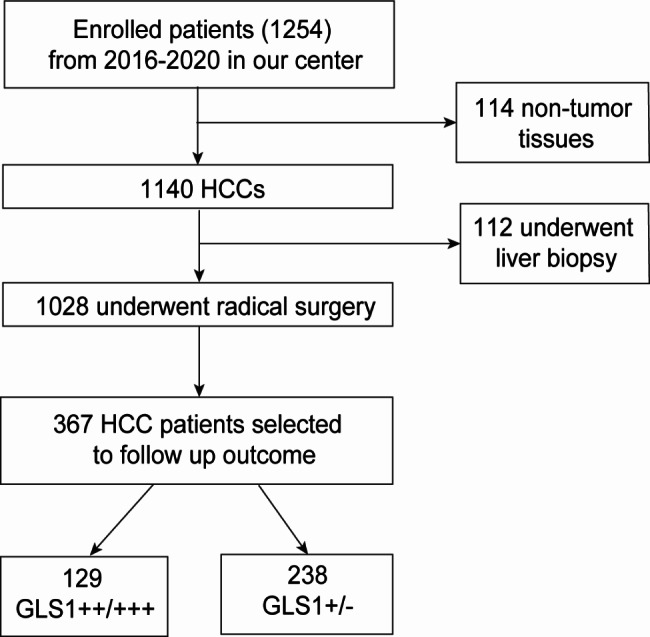
Flowchart of patient selection

Finally, 114 patients with nontumour tissues were enrolled, including 60 with focal hepatic hyperplasia, 14 with hepatic adenomas, and 40 with dysplastic nodules. Additionally, 1140 HCC patients were also enrolled and 1028 of them underwent radical surgeries within 5 years. The patient who underwent radical surgery had complete clinical and pathological data. The remaining patients who received liver biopsies were excluded (Fig. 1). All patients signed written informed consents. The registered clinical trial (ChiCTR-DDT-14,005,102, *chictr.org.cn*) was referred to for the exact protocol. After the radical surgery, many people were excluded in this study. The exclusion criteria were as follows: (1) with postoperative treatment, such as ablation, transarterial chemoembolization, chemotherapy, immunotherapy, or targeted therapy (530 patients); (2) didn’t have complete follow-up data (56); (3) didn’t received effective antiviral therapy (HBV/HCV, 75 patients). Finally, in this section, 367 HCC patients (GLS1-/+, 238; GLS1++/+++, 129) were incorporated to analyse the high-risk factors of HCC patients after radical surgery. They were reviewed by contrast-enhanced magnetic resonance imaging or computed tomography every 3 months and serum AFP every month.

### Magnetic resonance imaging examination

120 of the enrolled patients were received contrast-enhanced MRI examination (3.0T, uMR770, United Imaging, China) within two weeks before radical surgery. A T1-weighted volume interpolated breath-hold gradient recall echo sequence was used to provide an unenhanced scan, dynamic contrast-enhanced phase, and hepatobiliary phase (HBP). A dosage of 0.025 mmol/kg Gd-EOB-DTPA (Primovist; Bayer HealthCare, Berlin, Germany) was administered at a rate of 2.0 mL/s, and then followed by a 30-mL saline flush at the same rate. Using bolus triggering, a dual arterial phase sequence(AP) was started 15–25 s after the contrast media arrived at the distal thoracic aorta, while the portal venous phase (PP), delay phase (DP), and HBP were acquired at 1, 3, and 15 min after Gd-EOB-DTPA administration. The in-phase and out-phase T1 weighted images were used to define the steatosis or iron deposition in tumour and background liver. The apparent diffusion coefficient (ADC) was acquired by the diffusion-weighted imaging with b values of 0 and 800 s/mm^2^. The Liver Imaging Reporting and Data System scores were evaluated according to LI-RADS version 2018 [16]. All radiological results were obtained independently by two radiologists who were blind to other parts in consensus.

### Histopathological evaluation

The resected samples were fixed in 4% paraformaldehyde (pH 7.4, for 24 h), dehydrated, and embedded in paraffin. The nesting tissues were sectioned continuously at a thickness of 3.0 μm. The tissue slides were xylenated, hydrated in a series of alcohols, and then submerged in phosphate-buffered saline (PBS). The antigen retrieval was achieved in ethylene diamine tetraacetie acid (EDTA) buffer (pH 8.0, 100℃, 2.5 min). After washing with PBS, 100 μL of an endogenous peroxidase blocking agent (3% H_2_O_2_) was added and incubated for 10 min at room temperature. The slides were incubated with primary antibodies against human GLS1 (1:6400, Abcam, Hongkong, China, ab156876) for 60 min at 37℃. Next, they were treated with enzyme-labelled goat anti-mouse immunoglobulin G (IgG, ZSGB-BIO, Beijing, China, IB000087) for 30 min at 37℃. Then, 100 μL of diaminobenzidine (DAB) reagent (ZSGB-BIO, Beijing, China, IB000125) was added and incubated at room temperature for 6 min. The slides were washed with tap water, stained with hematoxylin staining solution for 30–60 s at room temperature, and differentiated with hydrochloric acid and alcohol until the tissues became blue. All experiments were performed by the same team. The operators were independent of the diagnostic results.

The degree of staining following the Fromowitz standard was used to determine the expression of GLS1, GPC3, AFP, antigen identified by monoclonal antibody Ki-67 (Ki67), and vascular endothelial growth factor receptor-2 (VEGFR2) [17]. In total, five randomized fields were evaluated and captured. According to the percentage of positively stained cells, the range scores depicting the number of stained cells were graded as follows: 0–5%, 6–15%, 16–50%, and ≥ 51%. The extent scores indicating staining intensity were given as “–“ (unstained, negative), “+” (brown stained, mildly positive), “++” (dark brown stained, moderately positive), or “+++” (darker brown stained, strongly positive). To sum up, the target protein expression status was assessed using the range and extent scores. The degree of steatosis in the background liver was estimated by referring to the nonalcoholic fatty liver disease activity score system (F) [18]. The Scheuer system was used to assess the degree of inflammation (G) and fibrosis (S) in the background liver [19]. In fact, all definitions in the present study were referred to the WHO guidelines.

### Receiver operating characteristic analysis

The crucial value of HCC diagnosis was determined using the receiver operating characteristic (ROC) curve. The cutoff value was determined using the maximum value of the Youden index. The results above the cutoff value were classified as positive, while the results below the cutoff value were deemed negative. The threshold value was judged as positive. The univariate and multivariate logistic regression analyses were carried out for important indicators predicting HCC. The “pROC” package was used to perform bootstrapping with 1000 iterations for the ROC analysis to validate the internal discrimination. Nomogram construction was completed with R software version 3.6.3 (https://www.r-project.org/) using “rms” packages. The level of over- or underestimation of projected probability compared with the observed probabilities of HCC was visually investigated using the calibration plot of the “rms” package, which was confirmed using 1000 rounds of bootstrapping internally. The “rmda” package was used to conduct decision curve analysis (DCA).

### Statistical analysis

Statistical analysis was conducted using SPSS 25.0 (IBM, Chicago, USA). The mean ± standard deviation or median (interquartile range, IQR) were used to present the data. The Kruskal–Wallis test or one-way analysis of variance was performed for continuous variables, and the chi-square tests or Fisher’s tests were conducted, if necessary, for categorical variables. The correlation test for the GLS1 expression with other parameters was performed with Kendall’s test. The log-rank test was used for the Kaplan–Meier survival analysis with R software. The Cox proportional-hazards regression model was used for the univariate analysis, and variables with a *P* < 0.1 were chosen for the multivariate analysis. The entry time was the date of surgery, while the exit time was the date of the first tumour recurrence/metastasis or the participant’s death for any reason (the last follow-up time for patients who are lost to follow-up; patients who were still alive on the end of follow-up date). The end of follow-up date was March 8, 2022. After careful calculation, the median follow-up time was 1049 days. The outcome was defined as the first tumour recurrence/metastasis. DFS referred to length from the entry time to exit time. All tests were two sided, and the statistical significance was judged to exist at a *P* < 0.05.

## Results

### Efficiency of GLS1 in hepatocellular carcinoma diagnosis

**Fig. 2:**
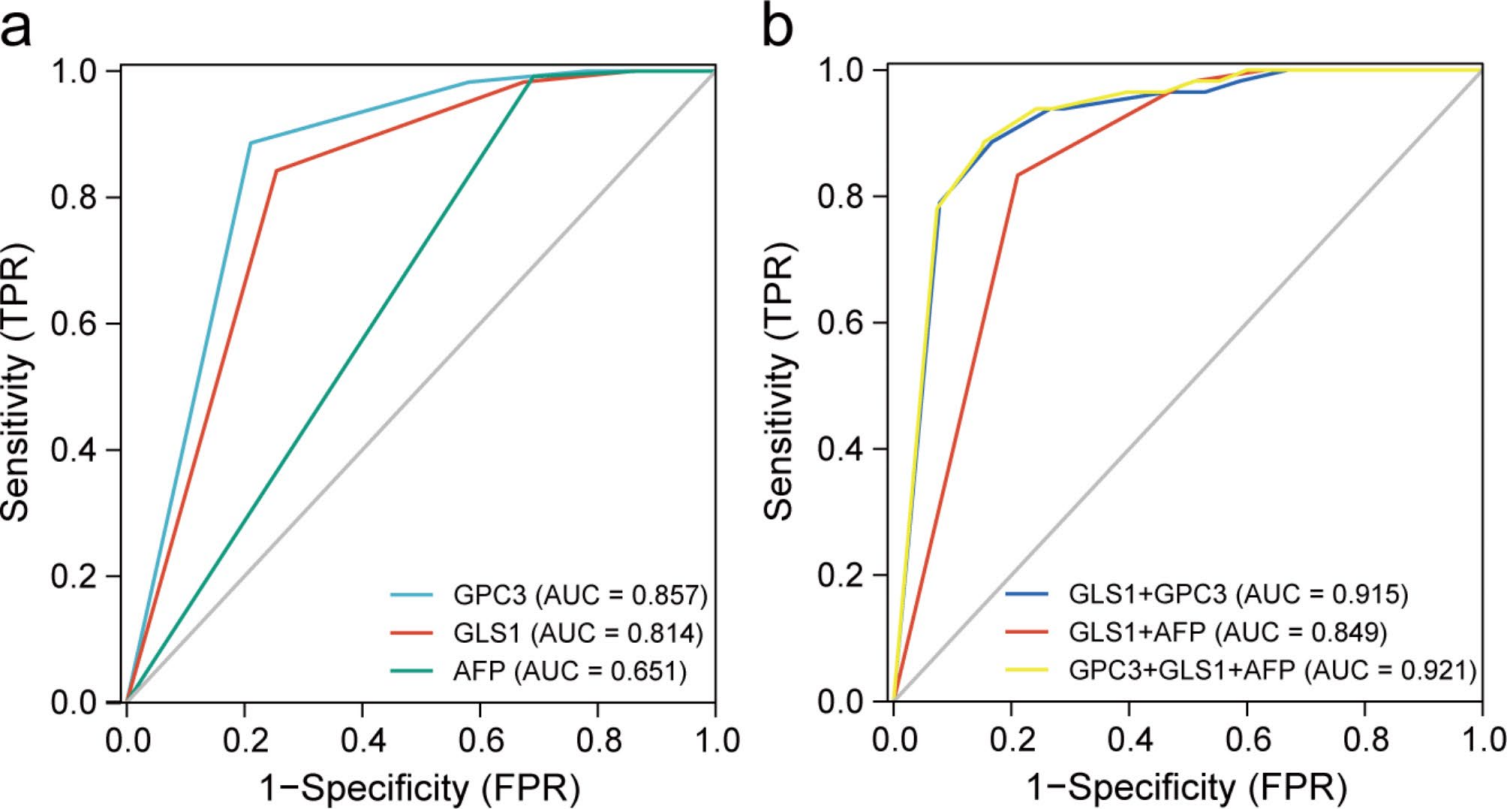
Sensitivity and specificity of the biomarker for HCC. (**a** and **b**) ROC curve analysis for the performance of the GPC3, GLS1, and AFP and their combination to diagnose HCC

In our respective study, we found that GLS1 expression was increased from displastic nodules to HCCs [15]. The expression of GLS1, GPC3, and AFP was detected using immunohistochemistry in enrolled patients. We determined HCC and nontumour tissues based on the optimal cutoff point determined by the ROC curve. The details are shown in Table 1. As shown in Table 1, the sensitivity (Sen), specificity (Spe), positive predictive value (PPV), negative predictive value (NPV), and Youden index of GLS1 diagnosing HCC were 0.746, 0.842, 0.979, 0.249, and 0.588, respectively. The value of GPC3 was 0.789, 0.886, 0.986, 0.296, and 0.675, while the value of AFP was 0.310, 0.991, 0.997, 0.126, and 0.310, respectively. The area under the curve (AUC) of GPC3 and AFP were 0.857 and 0.651, respectively (Fig. 2a). The diagnostic performance of GLS1 was between those of AFP and GPC3 (AUC value = 0.814, Fig. 2a).

**Table 1:** Diagnostic efficacy of GLS1, AFP, GPC3 and their combination for diagnosing HCC

variable	Sen	Spe	PPV	NPV	Youden index
GPC3	0.789	0.886	0.986	0.296	0.675
AFP	0.310	0.991	0.997	0.126	0.301
GLS1	0.746	0.842	0.979	0.249	0.588
GPC3 + GLS1	0.833	0.886	0.987	0.347	0.719
AFP + GLS1	0.789	0.833	0.979	0.284	0.623
GPC3 + GLS1 + AFP	0.846	0.886	0.987	0.366	0.732

Abbreviations: GPC3, glypican3; GLS1, Kidney-type glutaminase; AFP, alpha-fetoprotein; Sen, sensitivity; Spe, specificity; NPV, negative predictive value; PPV, positive predictive value

### Combination of GLS1 and GPC3 could achieve better diagnostic efficiency

**Fig. 3:**
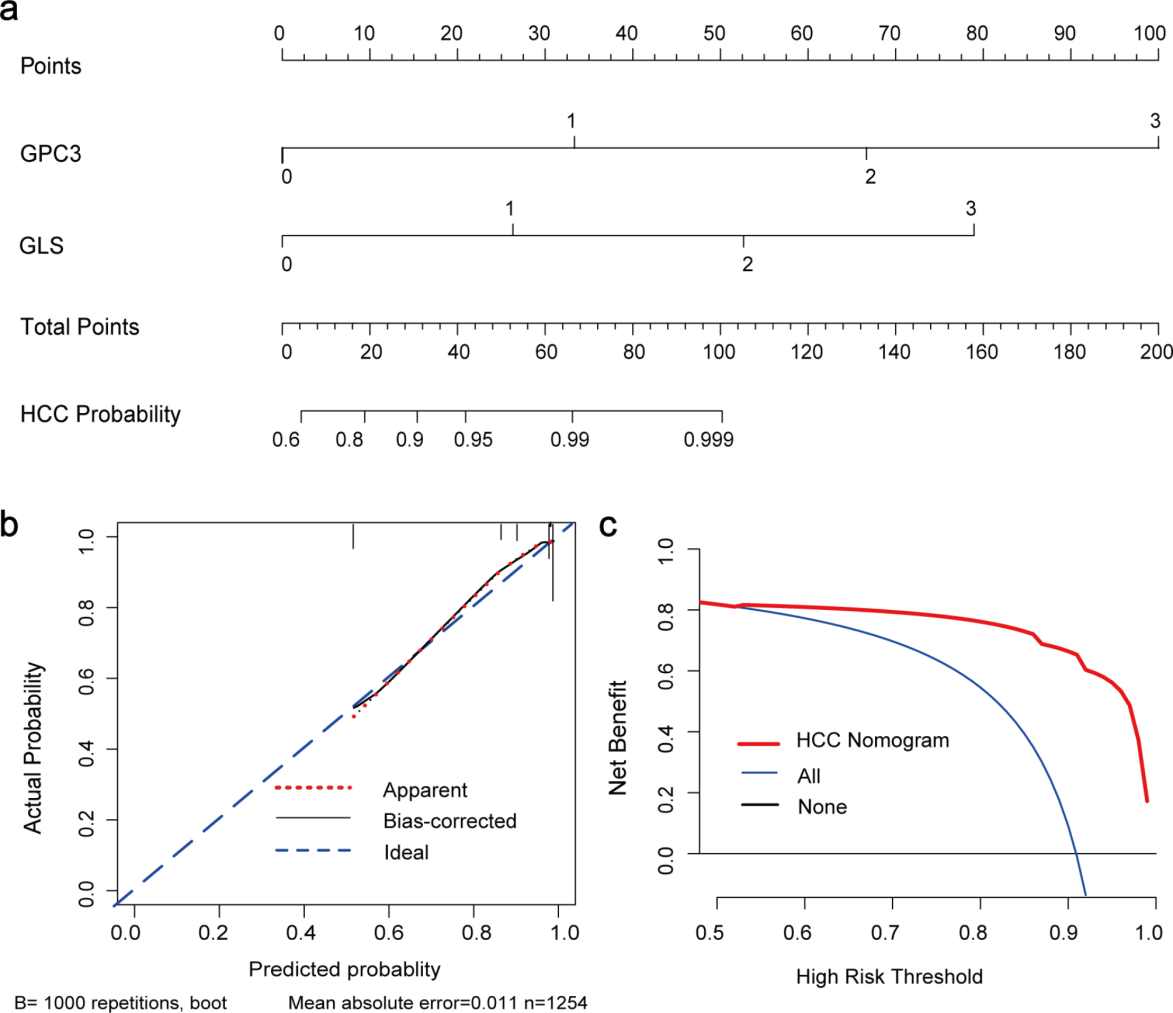
HCC-risk nomogram construction and validation. (**a**) Risk nomogram including GPC3 and GLS1 for predicting HCC. (**b**) Calibration curves depict the calibration of the nomogram in terms of the agreement between the predicted risk of HCC and actual HCC. The 45˚ blue line represents a perfect prediction, and the red dashed line represents the predictive performance of the nomogram, together with a bias-corrected black solid line. The closer the dashed line to the ideal line, the better the predictive accuracy of the nomogram. (**c**) Decision curve analysis for the HCC-risk nomogram. The red line represents the nomogram, the gray line represents the hypothesis that all patients have HCC, and the black line represents the hypothesis that no patients have HCC. The decision curve shows that if the threshold probability is between 0.54 and 1, then using the HCC-risk nomogram to predict HCC status adds more benefit than taking intervention to either all or no patients

We hoped that GLS1 not only might become an effective biological marker for diagnosing HCC but also might be combined with other indicators to increase its diagnostic performance. Also, the combined AUC value was higher. The Sen, Spe, PPV, NPV, and Youden index of GLS1 combined with AFP were 0.789, 0.833, 0.979, 0.284, and 0.623, respectively (Table 1; Fig. 2b). The value of GLS1 combined with AFP and GPC3 was 0.846, 0.886, 0.987,0.366, and 0.732, respectively (Table 1; Fig. 2b). The AUC value was 0.849 and 0.921, respectively (Fig. 2b). We searched for important HCC indicators. The univariate and multivariate logistic regression analyses revealed that GPC3 [odds ratio 11.4, 95% confidence interval (CI) 5.9–22.2, *P* < 0.001] and GLS1 (odds ratio 6.2, 95% CI 3.4–11.1, *P* < 0.001) were independent predictors for HCC (Supplemental Table 2). Furthermore, an HCC-risk nomogram internally validated by bootstrapping was established with these two predictors to show the joint diagnostic efficiency more visually (Fig. 3a). The nomogram discrimination was performed using ROC analysis, and the AUC was 0.915 (95% CI: 0.891–0.940) (Fig. 2b, the blue line). The specificity and sensitivity were 0.866 and 0.833, respectively. Moreover, the bootstrapped calibration curve of the nomogram, with a mean absolute error of 0.011, revealed no untoward deviation between the predicted risk and the actual risk of HCC (Fig. 3b). Further, the DCA was performed to investigate clinical benefits (Fig. 3c). The nomogram offered greater net benefits than imposing intervention to either all patients or none, with threshold probabilities ranging from 0.54 to 1.

### GLS1 expression was relevant to the HCC-related clinicopathological parameters

**Fig. 4:**
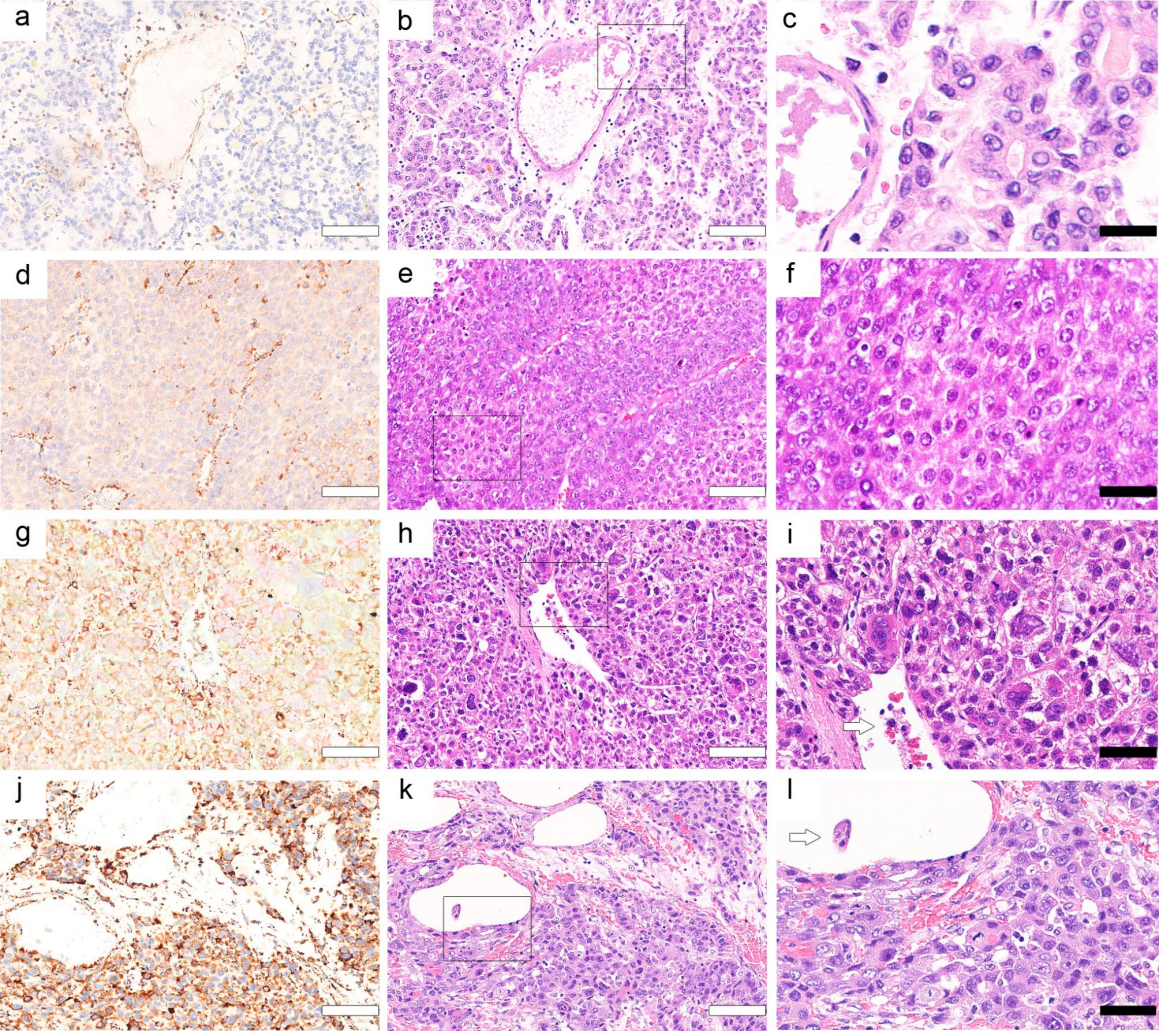
Relationship between GLS1 expression and Edmondson–Steiner grade or MVI among patients with HCC (**a**, **d**, **g**, and **j**). Immunohistochemistry staining of GLS1 in HCC defined as negative (–), weakly positive (+), moderately positive (++), and strongly positive (+++) (**b**, **e**, **h**, and **k**). Hematoxylin and eosin staining results of Edmondson–Steiner grade I, II, III, and III (**c**, **f**, **i**, and **l**). Magnification of the area indicated by the black square in (**b**, **e**, **h**, and **k**). MVI in (**i** and **l**) is shown with the black arrow. White bars = 250 μm; black bars = 100 μm

The clinicopathological features of the enrolled patients are summarized in Table 2. Following the careful pathological review, these patients were categorized into four groups according to the degree of immunohistochemistry expression: GLS1 negative (–), mild (+), moderate (++), and strong (+++). Further, 77 patients with HCC did not have VEGFR2 immunohistochemical results. Patients with moderate or strong GLS1 expression were confirmed as younger (*P* = 0.002) with greater probability of microvascular invasion (MVI) (*P* < 0.001) and higher Edmondson–Steiner grade, T stage (*P* = 0.019), TNM stage (*P* = 0.010), Ki67 expression (*P* < 0.001), VEGFR2 expression (*P* = 0.003), and GPC3 and AFP expression (all *P* < 0.001). The tumour dimension and GLS1 expression were correlated (*P* = 0.037) but not linearly (*P* = 0.729). We further explored the strength of the correlation. GLS1 was correlated with GPC3 (*r* = 0.202, *P* < 0.001) and AFP (*r* = 0.144, *P* < 0.001) expression, MVI (*r* = 0.112, *P* < 0.001, Fig. 4), Edmondson–Steiner grade (*r* = 0.195, *P* < 0.001, Fig. 4), TNM (*r* = 0.066, *P* = 0.017), T (*r* = 0.067, *P* = 0.018), Ki67 level (*r* = 0.170, *P* < 0.001), and VEGFR2 level (*r* = 0.099, *P* < 0.001). No statistically significant difference was observed in sex and F/G/S scores among different GLS1 groups. The relationship of GLS1 with Edmondson–Steiner grade and MVI is shown in Fig. 4. GLS1 was mildly negatively correlated with age at diagnosis in patients with HCC (*r* = − 0.071, *P* = 0. 002) (Table 2). During follow-up, GLS1 expression increased, and T stage and TNM stage were also higher. Patients with HCC having higher GLS1 expression were more likely to develop MVI and Ki67 higher expression. HCC had a stronger ability to proliferate and replicate in such patients.

**Table 2:** Clinicopathological parameters of the enrolled patients and their correlation with GLS1 expression level

characteristics	GLS1				p^*^	p’	r
		+	++	+++			
Age (years)	59.8 ± 10.6	58.7 ± 10.3	58.6 ± 10.4	55.8 ± 10.7	0.002	0.002	-0.071
Gender					0.746	0.280	0.03
Male	244 (84.1)	396 (82.8)	173 (80.8)	128 (81.0)			
Female	46 (15.9)	82 (17.2)	41 (19.2)	30 (19.0)			
Dimension (cm)	4.1 (2.8–6.5)	3.8 (2.3–6.5)	4.5 (2.6-8.0)	4.2 (2.5-7.0)	0.037	0.729	0.008
GPC3					< 0.001	< 0.001	0.202
	88 (30.5)	101(21.1)	26 (12.2)	24 (15.3)			
+	113 (39.1)	199 (41.6)	68 (31.9)	42 (26.8)			
++	50 (17.3)	93 (19.5)	59 (27.7)	27 (17.2)			
+++	38 (13.1)	85 (18.8)	60 (28.2)	64 (40.7)			
AFP					< 0.001	< 0.001	0.144
	239 (82.7)	313 (65.5)	136 (63.9)	97 (61.8)			
+	30 (10.3)	105 (22.0)	45 (21.1)	25 (16.0)			
++	10 (3.5)	36 (7.5)	18 (8.5)	17 (10.8)			
+++	10 (3.5)	24 (5.0)	14 (6.5)	18 (11.4)			
MVI					< 0.001	< 0.001	0.112
Negative	204 (76.4)	307 (70.4)	120 (63.2)	82 (60.7)			
Positive	63 (23.6)	129 (29.6)	70 (36.8)	53 (39.3)			
Edmondson-Steiner grade					< 0.001	< 0.001	0.195
I	30 (11.2)	25 (5.7)	8 (4.2)	3 (2.2)			
II	151 (56.6)	219 (50.2)	83 (43.7)	46 (34.1)			
III	79 (29.6)	185 (42.4)	87 (45.8)	73 (54.1)			
IV	7 (2.6)	7 (1.6)	12 (6.3)	13 (9.6)			
TNM stage					0.010	0.017	0.066
I	151 (56.5)	249 (57.1)	90 (47.4)	61 (45.2)			
II	67 (25.1)	136 (31.2)	62 (32.6)	48 (35.6)			
III-IV	49 (18.4)	51 (11.7)	38 (20.0)	26 (19.2)			
T stage					0.019	0.018	0.067
1	152 (56.9)	250 (57.3)	92 (48.4)	62 (45.9)			
2	67 (25.1)	137 (31.4)	63 (33.2)	48 (35.6)			
3	30 (11.2)	26 (6.0)	20 (10.5)	12 (8.9)			
4	18 (6.7)	23 (5.3)	15 (7.9)	13 (9.6)			
F					0.374	0.245	-0.033
0	180 (67.5)	321 (73.7)	140 (73.7)	98 (72.6)			
1	70 (26.2)	90 (20.6)	36 (18.9)	25 (18.5)			
2	14 (5.2)	20 (4.6)	12 (6.3)	9 (6.7)			
3–4	3 (1.1)	5 (1.1)	2 (1.1)	3 (2.2)			
G					0.089	0.399	-0.023
0	7 (2.6)	11 (2.5)	2 (1.1)	1 (0.7)			
1	88 (33.0)	141 (32.3)	56 (29.5)	54 (40.0)			
2	107 (40.1)	211 (48.4)	90 (47.4)	63 (46.7)			
3–4	65 (24.3)	73 (16.7)	42 (22.1)	17 (12.6)			
S					0.551	0.873	0.004
0	31 (11.6)	44 (10.1)	12 (6.3)	9 (6.7)			
1	38 (14.2)	80 (18.4)	26 (13.7)	20 (14.8)			
2	29 (10.9)	58 (13.3)	35 (18.4)	29 (21.5)			
3	45 (16.9)	69 (15.8)	31 (16.3)	19 (14.0)			
4	124 (46.4)	185 (42.4)	86 (45.3)	58 (43.0)			
Ki67					< 0.001	< 0.001	0.170
	32 (12.0)	32 (7.3)	14 (7.3)	6 (4.4)			
+	105 (39.3)	138 (31.7)	48 (25.3)	29 (21.5)			
++	116 (43.4)	229 (52.5)	103 (54.2)	72 (53.3)			
+++	14 (5.4)	37 (8.5)	25 (13.2)	28 (20.8)			
VEGFR2					0.003	< 0.001	0.099
	102 (43.0)	161 (39.7)	58 (33.1)	36 (27.5)			
+	93 (39.2)	157 (38.7)	77 (44.0)	54 (41.2)			
++	31 (13.0)	64 (16.7)	29 (16.6)	29 (22.1)			
+++	11 (4.6)	24 (5.9)	11 (6.7)	12 (9.2)			

The p’ was the value of Kendall’s test. The p* was the value of the Kruskal-Wallis test, Chi-square tests and one-way analysis of variance. Dimension are presented as median (interquartile range, IQR). Age is presented as mean ± SD (standard deviation), while categorical variables are presented as patients (%)

Abbreviations: GPC3, glypican3; GLS1: Kidney-type glutaminase; AFP: alpha-fetoprotein; Ki67, antigen identified by a monoclonal antibody; MVI, microvascular invasion, F, the degree of steatosis in the background liver; G, the degree of inflammation in the background liver; S, degree of fibrosis in the background liver; VEGFR2, vascular endothelial growth factor receptor-2; Ki67, antigen identified by monoclonal antibody Ki-67

### GLS1 expression was relevant to the radiological parameters

Table 3 provides a summary of the relationship between MRI-related parameters and GLS1 expression in the tumour. We defined GLS1-/+ as negative expression (n = 81) and GLS1++/+++ as positive expression (n = 31). Patients positive for GLS1 were confirmed to have lower LI-RADS scores (*P* = 0.026), lower proportion of nonrim arterial phase hyperenhancement (*P* = 0.004) and nonperipheral washout in PP or DP (*P* = 0.020). Besides, the lesion-to-liver signal ratio in arterial phase (*P* = 0.038), portal veinous phase (*P* = 0.040), delay phase (*P* = 0.002) and hepatobiliary phase (*P* < 0.001) were validated higher in the GLS1-positive group. Thirty-two HCC patients have also undergone positron emission tomography / computed tomography examinations (PET/CT) at the same time. But there was no statistically significant difference in the maximum standard uptake value of ^1^^8^F-glucose.

**Table 3:** The relationships between radiological characteristics and GLS1 expression level

Ce-MRI characteristics	n = 120	GLS1		*P* value
		Negative (-/+, n = 89)	Positive (++/+++, n = 31)	
Serum AFP (ng/ml)	20.85 (2.93−131.58)	14.00 (2.85−115.55)	52.40 (3.40−226.50)	0.118
Dimension (cm)	3.35 (2.20–5.17)	3.40 (2.45–5.10)	2.80 (2.00−5.60)	0.549
Tumor in vein (+)	5 (4.2)	3 (3.4)	2 (6.5)	0.603
Shape				0.053
Round	85 (70.8)	68 (76.4)	17 (54.8)	
Lobular	9 (7.5)	5 (5.6)	4 (12.9)	
Irregular	26 (21.7)	16 (18.0)	10 (32.3)	
Nonrim APHE (+)	92 (76.7)	74 (83.1)	18 (58.1)	**0.004**
Nonrim APHE (-)	28 (23.3)	15 (16.9)	13 (41.9)	
Nonperipheral washout (+)	95 (79.2)	75 (84.3)	20 (64.5)	**0.020**
Nonperipheral washout (-)	25 (20.8)	14 (15.7)	11 (35.5)	
Enhancing capsule (+)	19 (15.8)	15 (16.9)	4 (12.9)	0.778
Blood in mass (+)	12 (10)	8 (9)	4 (12.9)	0.532
Fat in mass (+)	51 (42.5)	40 (44.9)	11 (35.5)	0.359
Necrosis (+)	11 (9.2)	8 (9.0)	3 (9.7)	0.909
Maxium ADC (×10 ^− 6^ mm^2^ /s)	1031 (868–1255)	1076 (874–1255)	974 (862–1281)	0.299
Minium ADC (×10 ^− 6^ mm^2^ /s)	651 (516–790)	663 (542–832)	622 (463–737)	0.150
Median ADC (×10 ^− 6^ mm^2^ /s)	817 (713–993)	835 (714–1013)	785 (641–890)	0.184
Liver ADC (×10 ^− 6^ mm^2^ /s)	1022 (919–1054)	1012 (935–1153)	1055 (894–1163)	0.129
Lesion-to-liver ADC ratio	0.821 (0.702–0.935)	0.837 (0.717–0.951)	0.783 (0.678–0.875)	0.074
Satellite nodules (+)	28 (23.5)	21 (23.9)	7 (22.6)	0.885
Liver background				0.243
Fat	38 (31.7)	31 (34.8)	7 (22.6)	
Iron	12 (10.0)	7 (7.9)	5 (16.1)	
LIRADS v2018 category				**0.026**
LIRADS−3	18 (15.0)	9 (10.1)	9 (29.0)	
LIRADS−4	32 (26.7)	23 (25.8)	9 (29.0)	
LIRADS−5	70 (58.3)	57 (64.0)	13 (41.9)	
Lesion-to-liver ratio (AP)	1.231 (0.987–1.580)	1.301 (1.010–1.627)	1.108 (0.850–1.352)	**0.038**
Lesion-to-liver ratio (PP)	0.847 (0.699–0.930)	0.857 (0.735–0.954)	0.752 (0.611–0.884)	**0.040**
Lesion-to-liver ratio (DP)	0.733 (0.620–0.833)	0.774 (0.662–0.852)	0.654 (0.549–0.738)	**0.002**
Lesion-to-liver ratio (HBP)	0.530 (0.423–0.659)	0.568 (0.456–0.683)	0.423 (0.369–0.493)	**< 0.001**
Lesion-to-liver ratio in uncontrast-enhanced T1	0.764 (0.658–0.898)	0.776 (0.658–0.904)	0.714 (0.660–0.885)	0.528
Lesion-to-liver ratio (T2)	2.096 (1.707–2.591)	2.096 (1.741–2.591)	1.976 (1.654–2.639)	0.735
Eligible observations	32	16	16	
SUVmax	3.75 (2.90–6.90)	3.35 (2.80–5.23)	5.15 (3.10–8.40)	0.080

The continuous variables are presented as median (interquartile range, IQR), while categorical variables are presented as patients (%). The P value was acquired by the U test and chi-square test

Abbreviations: APHE: arterial phase hyperenhancement, ADC: apparent diffusion coefficient, AFP: alpha-fetoprotein, AP: arterial phase, PP: portal veinous phase, DP: delay phase, HBP: hepatobiliary phase, SUVmax: maximum standard uptake value

### GLS1 predicted the postoperative recurrence of patients with HCC

**Fig. 5:**
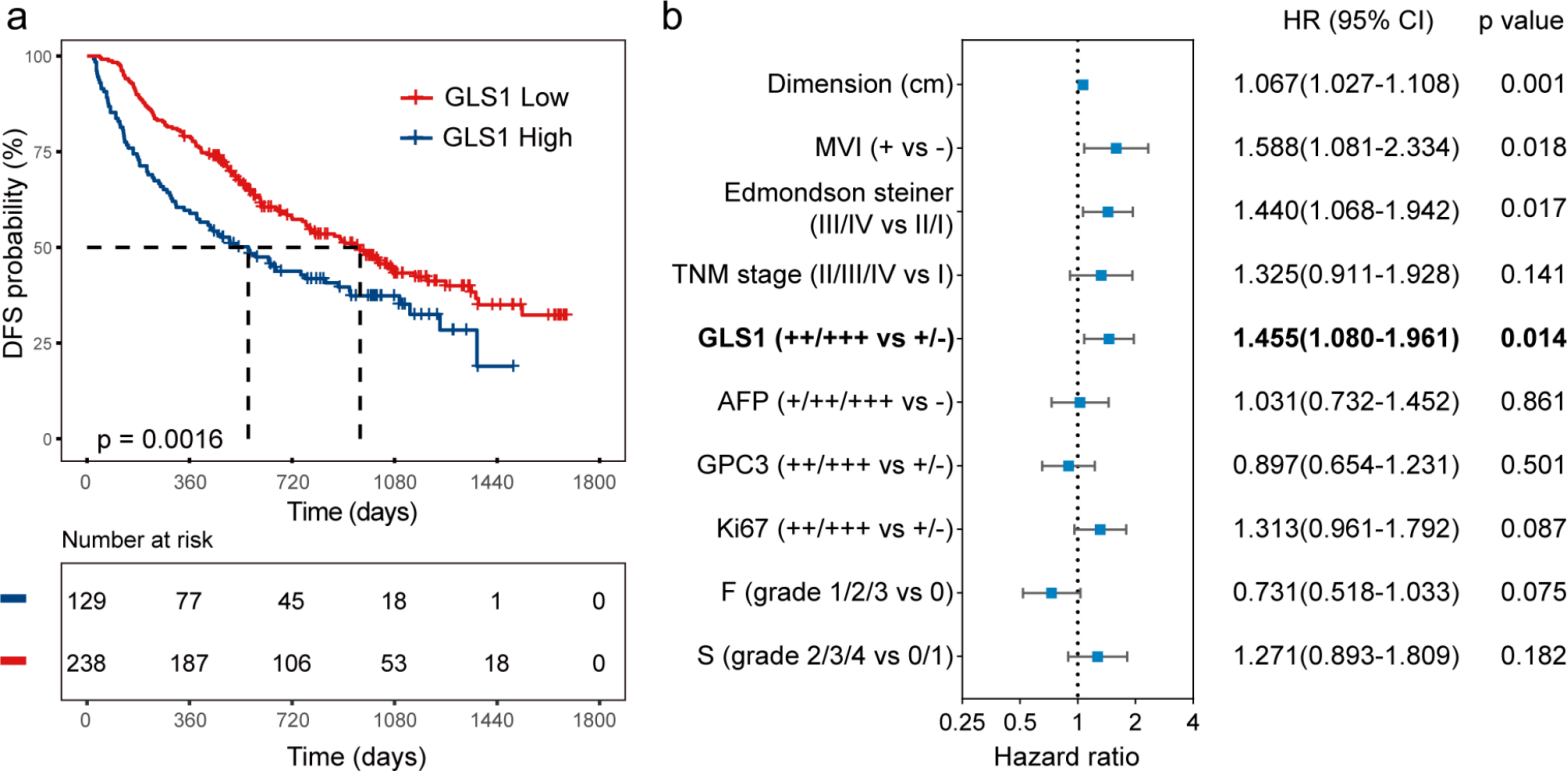
Relationship between different GLS1 expression and DFS after radical resection in patients. (**a**) DFS of the groups stratified by GLS1. Multivariate Cox analysis confirmed the independent prognostic value of GLS1 expression (**b**)

In our retrospective study, we explored the relationship between GLS1 and overall survival (OS), indicating that GLS1 was a prognostic biomarker for patients with HCC [15]. The high expression of GLS1 indicated a poor prognosis. However, we did not explore the relation between GLS1 and DFS. Further, 367 patients with HCC who underwent radical resection were selected to observe the recurrence. We divided them into GLS1 low-expression (-/+, 238 patients) and high-expression (++/+++, 129 patients) groups. As shown in Fig. 5a, GLS1 was significantly associated with DFS (*P* = 0.016, Fig. 5a) after radical resection in patients with HCC. The gaps were statistically significant. We also attempted to use Cox regression model to assess the independent predictive value of GLS1 expression. The baseline variables associated with DFS in the univariate Cox regression analysis were dimension (HR = 1.094, 95% CI 1.058–1.132, *P* < 0.001), TNM stage (HR = 2.222, 95% CI 1.675–2.949, *P* < 0.001), MVI (HR = 2.551, 95% CI 1.932–3.369, *P* < 0.001), Edmondson–Steiner grade (HR = 1.595, 95% CI 1.206–2.110, *P* = 0.001), GLS1 (HR = 1.568, 95% CI 1.184–2.077, *P* = 0.002), GPC3 (HR = 1.319, 95% CI 1.001–1.739, *P* = 0.049), AFP (HR = 1.316, 95% CI 0.979–1.770, *P* = 0.069), Ki67 (HR = 1.759, 95% CI 1.331–2.324, *P* < 0.001) (Supplemental Table 3), F (HR = 0.620, 95% CI 0.444–0.866, *P* = 0.005), and S (HR = 1.357, 95% CI 0.970–1.897, *P* = 0.074). The multivariate analysis confirmed the independent prognostic value of GLS1 expression (HR = 1.455, 95% CI 1.080–1.961, *P* = 0.014) (Fig. 5b and Supplemental Table 3).

## Discussion

On the one hand, the present study explored the efficacy of GLS1 in diagnosing HCC and its correlation with the biological behavior of HCC. On the other hand, we found that GLS1 was associated with DFS after radical resection among HCC patients and some radiological parameters.

Most HCCs develop on the basis of cirrhotic nodules. Therefore, it is necessary to identify accurate biomarkers to distinguish early-stage HCCs from the cirrhotic nodules. The proper definition of the lesion improve the diagnostic accuracy, which can help the patients receive prompt treatment and achieve better survival. In recent years, researchers have been trying to find specific markers for the early diagnosis of HCC, thereby improving the prognosis of patients with HCC. At present, few studies have explored GLS1 as a biological marker for diagnosing HCC. Especially studies on its correlation with clinicopathological parameters are lacking.

GPC3, a member of the glypican family, is highly expressed in large part of HCCs and regarded as a specific diagnostic marker for HCC [20, 21]. But it was not related to the DFS of HCC patients (Fig. 5b). Our results showed that the diagnostic performance of GLS1 was close to that of GPC3 and better than that of AFP. AFP is one of the most widely used serological markers for HCC, but its sensitivity and specificity are not perfect due to the elevation of its levels in patients with cirrhosis and active hepatitis [22, 23]. In our respective study, we found that GLS1 was an independent diagnostic factor with high sensitivity and specificity for HCC. GLS1 may be essential for the advancement of liver malignancies due to its high hydrolytic efficiency. Additionally, our prior research established a correlation between the expression of GLS1 and c-Myc which could stimulate the cell proliferation and upregulate glutamine catabolism in mitochondria [14, 24–26]. We further validated the good diagnostic performance of GLS1, and it was no better than our previous findings. We analyzed that it might be due to more biases in retrospective studies; the conclusions drawn from prospective large-sample studies might be more convincing. We confirmed that GLS1 combined with GPC3 and AFP could improve the diagnostic performance of each alone, resulting in a higher AUC. This was consistent with the findings of Xu et al. [27]. We further established and validated a predictive model through logistic regression, achieving clinical benefits.

Further, we explored the correlation between GLS1 and clinical data or tumour biological behaviour. Higher expression of GLS1 meant the higher expression of AFP, GPC3, Ki67, and VEGFR2; higher T and TNM stages; and higher Edmondson–Steiner grade, which indirectly reflected the relationship between GLS1 and tumour proliferation and invasion ability. This was largely confirmed by Xu et al. [13]. Microvascular invasion and Ki67 are considered to be two key factors for the short-term recurrence and prognosis of HCC after surgery [28–30]. At present, specific inhibitory of GLS1, such as Bis-2-[5-(phenylacetamido)-1,3,4-thiadiazol-2-yl]ethyl sulfide (BPTES) or its analogs (for example, N-(5– [1, 3, 4]thiadiazol-2-yl)-2-phenyl-acetamide 6) had been used as molecular probes to justify the efficacy of GLS1 inhabitory in many cancers [31, 32]. BPTES, the inhabitor of KEG, promotes the development of an inactive complex and prevents the allosteric activation brought on by phosphate binding [33]. In an immune-competent MYC-mediated mouse model of HCC, treatment with BPTES could achieve better survival. BPTES also reduce the replication of DNA and inhibit the growth of tumor cells [34]. Given that GLS1 had a clinical application significance, we also tried to assess GLS1 expression in tumors with MRI features, hoping to provide more information before surgery. If we could better predict the GLS1 expression of HCC patients, we may have the potential to select sensitive populations for GLS1 inhibitors. In the present results, we found the correlation between GLS1 and radiological indexes, such as nonrim APHE, nonperipheral washout in portal venous phase, lesion-to-liver ratio in artery/portal venous/delay phase, etc. We hoped for more research with larger sample size to support the relationship between GLS1 expression and radiological parameters.

Additionally, we were the first to examine the relationship between GLS1 and DFS in patients with HCC after radical hepatectomy. The subgroup analysis indicated that elevated GLS1 expression could predict poorer DFS (*P* = 0.0016) after radical surgeries. Although our findings were encouraging and statistically significant, we are yet unsure of the precise causes for the variations in survival among patients with HCC having different levels of GLS1 expression. The higher the GLS1 expression, the more active the glutamine metabolized in mitochondria, and the stronger the tumour cell metabolism, leading to poor prognosis in patients with HCC [11, 35]. The increase in GLS1 expression was the cause, and the subsequent increase in other indicators was the effect. We only showed the link between GLS1 expression and prognosis, but the mechanism deserves further exploration.

Several limitations of this prospective study should be considered. First, the sample size of benign liver masses enrolled in this study was small and the proportion was inappropriate, leading to some bias in calculating diagnostic accuracy. Simultaneously, we only conducted internal invalidation of the nomogram. Second, since this study was consistent with previous retrospective study, its novelty was somewhat weakened. Third, all enrolled patients were from China, and we did not include all kinds of liver neoplasms. The generalization of our research findings globally is worthy of further exploration. Fourth, we did not examine the relationship between GLS1 and OS, as we considered the differences in the treatment options and responses of patients after recurrence. Additionally, we did not stratify the HCC into early-age and advanced HCC and cannot illustrate the diagnostic value better. Finally, all patients enrolled in this study had positive GLS1 expression in their tissues; however, the serum GLS1 expression levels were more readily detectable using a noninvasive technique. Whether serum-based GLS1 expression levels are more suitable for assessment has to be determined. We should also incorporate more confounding variables into our logistic regression, which may lead to a more valuable diagnostic model combined clinical and pathological characteristics.

## Conclusions

In conclusion, our study showed that GLS1 was overexpressed in HCC and correlated with poor DFS in patients with HCC. We also discovered the correlation between GLS1 expression and age, Ki67 and VEGFR2 expression, Edmondson–Steiner grade, MVI, T and TNM stage in HCC. GLS1 expression might offer significant data on HCC diagnosis and prognosis for guiding clinical therapy alone or in combination with other biomarkers. Based on previous findings, it was presumed that targeted glutaminase could inhibit cancer progression [32, 36, 37]. Hence, targeted glutaminase therapy may become an effective approach for treating HCC in the future. More prospective clinical research is urgently needed to validate its safety and effectiveness.

### Electronic supplementary material

Below is the link to the electronic supplementary material.

Supplementary Material 1

## Data Availability

All data and materials supporting our fndings can be obtained from cor responding author upon request. Data were private for the sake of privacy and ethical restrictions.

## Abbreviations

AFP: Alpha-fetoprotein
AP: Arterial phase
APHE: Arterial phase hyperenhancement
AUC: Area under ROC curve
DC: Apparent diffusion coefficient
DFS: Disease-free survival
DP: Delay phase
F: The degree of steatosis in the background liver
FPR: False-positive rate
G: The degree of inflammation in the background liver
GLS1: Kidney-type glutaminase
GPC3: Glypican3
HBP: Hepatobiliary phase
HCC: Hepatocellular carcinoma
Ki67: Antigen identified by a monoclonal antibody Ki67
MVI: Microvascular invasion
NPV: Negative predictive value
PP: Portal veinous phase
PPV: Positive predictive value
S: Degree of fibrosis in the background liver
Sen: Sensitivity
Spe: Specificity
SUVmax: Maximum standard uptake value
TPR: True-positive rate
VEGFR2: Vascular endothelial growth factor receptor-2
