# Supplementary material for: Kidney-type glutaminase is a biomarker for the diagnosis and prognosis of hepatocellular carcinoma: a prospective study
Source: BMC Cancer. 2023 Nov 9;23:1081. doi: 10.1186/s12885-023-11601-y (PMC10633901; doi:10.1186/s12885-023-11601-y)
Supplement: Supplementary file 1 — Supplementary Material 1 [file 12885_2023_11601_MOESM1_ESM.docx]

**Supplemental Table 1**: The number of patients classified as HCC and N-HCC according to biomarker intensity

|  |  | GPC3+ | GPC3- | GLS1+ | GLS1- | AFP+ | AFP- |
| --- | --- | --- | --- | --- | --- | --- | --- |
| Gold standard | HCC | 900 | 240 | 850 | 290 | 353 | 787 |
|  | N-HCC | 13 | 101 | 18 | 96 | 1 | 113 |

**Supplemental Table 2**: Univariate and multivariate logistic regression analyses for the prediction of HCC

| variable | univariate | | multivariate | |
| --- | --- | --- | --- | --- |
|  | OR (95%CI) | p | OR (95%CI) | p |
| GPC3 | 16.1 (8.5-30.7) | <0.001 | 11.4 (5.9-22.2) | <0.001 |
| GLS1 | 9.9 (5.7-17.1) | <0.001 | 6.2 (3.4-11.1) | <0.001 |
| AFP | 29.6 (4.1-213.1) | 0.01 | 7.8 (1.0-60.3) | 0.275 |

**Supplemental Table 3**: Univariate and multivariate Cox regression analyses for the DFS of HCC

| variable | Univariate regression analysis | | Multivariate regression analysis | |
| --- | --- | --- | --- | --- |
|  | HR (95% CI) | p value | HR (95% CI) | p value |
| Age (year) | 0.992 (0.980-1.004) | 0.209 |  |  |
| Gender (Female vs male) | 1.024 (0.724-1.448) | 0.894 |  |  |
| Dimension (cm) | 1.094 (1.058-1.132) | <0.001 | 1.067 (1.027-1.108) | 0.001 |
| Focality (multiple vs single) | 1.307 (0.936-1.825) | 0.115 |  |  |
| TNM (II/III/IV vs I) | 2.222 (1.675-2.949) | <0.001 | 1.325 (0.911-1.928) | 0.141 |
| MVI (+/-) | 2.551 (1.932-3.369) | <0.001 | 1.588 (1.081-2.334) | 0.018 |
| Edmondson-Steiner grade (III/IV vs II/I) | 1.595 (1.206-2.110) | 0.001 | 1.440 (1.068-1.942) | 0.017 |
| F (grade 1/2/3 vs 0) | 0.620 (0.444-0.866) | 0.005 | 0.731 (0.518-1.033) | 0.075 |
| G (grade 2/3/4 vs 0/1) | 1.194 (0.892-1.599) | 0.233 |  |  |
| S (grade 2/3/4 vs 0/1) | 1.357 (0.970-1.897) | 0.074 | 1.271 (0.893-1.809) | 0.182 |
| GLS1 (++/+++ vs +/-) | 1.568 (1.184-2.077) | 0.002 | 1.455 (1.080-1.961) | 0.014 |
| GPC3 (++/+++ vs +/-) | 1.319 (1.001-1.739) | 0.049 | 0.897 (0.654-1.231) | 0.501 |
| AFP (+/++/+++ vs -) | 1.316 (0.979-1.770) | 0.069 | 1.031 (0.732-1.452) | 0.861 |
| Ki67 (++/+++ vs +/-) | 1.759 (1.331-2.324) | <0.001 | 1.313 (0.961-1.792) | 0.087 |
